# The effectiveness and safety of sacubitril/valsartan in real-world dialysis patients with heart failure reduced ejection fraction

**DOI:** 10.1097/MD.0000000000046866

**Published:** 2026-01-30

**Authors:** Kuan-Chieh Tu, Yu-Chieh Weng, Chia-Te Liao, Jheng-Yan Wu

**Affiliations:** a Division of Cardiology, Department of Internal Medicine, Chi Mei Medical Center, Tainan, Taiwan; b School of Medicine, College of Medicine, National Sun Yat-sen University, Kaohsiung City, Taiwan; c Department of Nutrition, Chi Mei Medical Center, Tainan, Taiwan; d Department of Public Health, College of Medicine, National Cheng Kung University, Tainan City, Taiwan.

**Keywords:** advanced chronic kidney disease, end-stage kidney disease, heart failure reduced ejection fraction, sacubitril/valsartan

## Abstract

**Backgrounds:**

Sacubitril/valsartan (angiotensin receptor neprilysin inhibitor [ARNi]) effectively treats heart failure with reduced ejection fraction. Its impact on advanced chronic kidney disease (CKD) or end-stage renal disease (ESRD) remains unclear due to their exclusion from major trials. We aimed to evaluate ARNi’s effectiveness and safety in these populations.

**Methods:**

We systematically searched PubMed, Cochrane, Embase, and ClinicalTrials.gov for observational studies of ARNi use in adults with advanced CKD or ESRD requiring maintenance dialysis. Primary outcomes were all-cause mortality and heart failure hospitalization (HHF), with secondary outcomes focusing on left ventricular ejection fraction (LVEF), blood pressure, and biomarkers. A random-effects model was used to derive pooled estimates, and study quality was assessed using the Newcastle-Ottawa scale.

**Results:**

Ten observational studies included 4329 patients with advanced CKD or ESRD. The pooled odds ratio (OR) for mortality plus HHF was 0.54 (95% confidence interval [CI]: 0.25–1.18, *P* = .12). Subgroup analysis showed ORs of 0.67 (95% CI: 0.17–2.70) for ESRD and 0.64 (95% CI: 0.25–1.66) for CKD. The pooled OR for mortality alone was 0.86 (95% CI: 0.50–1.46, *P* = .57). In ESRD, ARNi was linked to significant LVEF improvement (+4.20%, *P* < .001) and reduced systolic blood pressure (−7.13 mm Hg, *P* = .008), without increased risk of hyperkalemia or hypotension.

**Conclusion:**

In advanced CKD and ESRD with heart failure with reduced ejection fraction, ARNi did not significantly reduce mortality or HHF but showed potential benefit in LVEF improvement. Larger randomized trials are required to confirm its efficacy and safety in this high-risk population.

## 1. Introduction

Heart failure affects millions of people worldwide every year and significantly increases healthcare expenditures.^[1]^ Recommended treatments for patients with heart failure with reduced ejection fraction (HFrEF) include drugs such as angiotensin-converting enzyme inhibitors, beta-blockers, and mineralocorticoid receptor antagonists. In addition, sacubitril-valsartan, an emerging medication classified as an angiotensin receptor neprilysin inhibitor (ARNi), has also been demonstrated to reduce major adverse cardiovascular events (MACE) among patients with HFrEF, as endorsed by the American Heart Association and the European Society of Cardiology.^[2,3]^

Excessive activation of the renin-angiotensin-aldosterone system (RAAS) is the main mechanism triggering and exacerbating the clinical presentation of heart failure.^[4]^ Natriuretic peptides, serving as compensatory neurohormones in heart failure, act on the kidney’s collecting duct system, playing a crucial role in regulating body fluid balance.^[5]^ Nevertheless, natriuretic peptides are subject to degradation by neprilysin, a neutral endopeptidase distributed throughout various parts of the body, including the kidneys, lungs, brain, endothelium, and heart.^[6]^ ARNi can effectively block both the RAAS and neprilysin, leading to an improvement in heart function and a reduction in myocardial remodeling. Ultimately, this dual action results in better clinical outcomes.^[7]^

In the PARADIGM trial, ARNi was shown to significantly reduce MACEs in patients with HFrEF.^[8]^ Moreover, accumulating evidence suggests that ARNi effectively lowers blood pressure in patients with hypertension.^[9,10]^ Studies also indicate that ARNi has great potential in the treatment of arrhythmia, myocardial infarction, diabetes mellitus, and diabetic nephropathy.^[11–14]^ However, it’s worth noting that large randomized controlled trials (RCTs) involving ARNi did not include patients with an estimated glomerular filtration rate (eGFR) below 30 mL/min/1.73 m^2^. Hence, the impact of this drug on patients with advanced chronic kidney disease (CKD; stage IV and stage V), or even those with end-stage renal disease (ESRD), can only be assessed through observational studies.^[15–17]^ Several observational studies have reported improvements in echocardiography parameters and N-terminal pro–B-type natriuretic peptide (NT-proBNP) levels in patients with HFrEF and advanced CKD/ESRD treated with ARNi. However, only a limited number of studies have investigated clinical outcomes, such as cardiovascular death and heart failure hospitalizations (HHFs), in this population. The question of whether ARNi is beneficial for patients with HFrEF and advanced CKD/ESRD remains a topic of debate, and therefore, to fill this knowledge gap, we conducted a systematic review of the available literature to assess the effectiveness and safety of ARNi on patients with advanced CKD and ESRD who are undergoing maintenance dialysis.

## 2. Materials and methods

This meta-analysis protocol was registered in International Prospective Register of Systematic Reviews (CRD420250651741) and followed the guidelines of the Preferred Reporting Items for Systematic Reviews and Meta-Analyses statement and Cochrane methods.

### 2.1. Databases and inclusion/exclusion criteria

#### 2.1.1. Strategy of literature search

A systematic review was conducted in PubMed, Cochrane, Embase, and ClinicalTrials.gov from the inception dates to February 2025 without restriction on language or geographic locations. The strategy consisted of the following medical subject headings terms: “heart failure,” “heart failure, systolic,” “heart failure, diastolic,” “renal insufficiency,” “chronic renal insufficiency,” “ESRD,” “chronic renal disease,” “CKD,” “dialysis,” “hemodialysis,” “renal dialysis,” “sacubitril,” “neprilysin inhibitor,” “valsartan,” “angiotensin receptor blocker” or “Entresto” or “valsartan sacubitril.” Two investigators (YCW and KCT) independently screened observational studies and selected full-texts for quality assessment and data synthesis. Authors of included articles were contacted for missing data.

#### 2.1.2. Inclusion and exclusion criteria

Studies were eligible if they involved adults (≥18 years) with advanced CKD (eGFR < 30 mL/min/1.73 m^2^), or ESRD on dialysis, included an ARNi group, used a placebo or had no control group, and reported all-cause mortality, HHF, and/or differences in left ventricular ejection fraction (LVEF), systolic blood pressure (SBP), diastolic blood pressure (DBP), left ventricular end-diastolic dimension (LVEDD), left atrial diameter (LAD), or NT-proBNP. Studies with data only in abstracts were included if sufficient information could be extracted. Exclusions were patients <18 years, unavailable outcome data, and reviews, letters, case reports, or non-original research. Two authors (KCT, YCW) independently screened titles/abstracts and resolved any disagreements by discussion.

### 2.2. Data extraction

For each eligible study, general information (1st author, year of publication, study name, study design, sample size), baseline demographic and clinical characteristics of the participants (population, age, percentage of male, kidney replacement therapy [KRT] modality, comorbidities, follow-up duration), interventions/exposure (ARNi), effectiveness outcome data (e.g., HHF, all-cause mortality, LVEF, LVEDD, LAD, NT-proBNP, SBP, and DBP) and safety outcome data (e.g., risk of hyperkalemia, hypotension and progression to dialysis) were extracted. In cases where the full article was not accessible, we extracted available data from the abstracts of the relevant studies. Additionally, if the articles did not provide original data but presented their findings graphically, we employed the WebPlotDigitizer program to extract and reconstruct the original data points from these graphs. This approach allowed us to obtain and analyze the necessary data for our meta-analysis, ensuring a thorough evaluation of the impact of ARNi on patients with advanced CKD and ESRD undergoing maintenance dialysis.

### 2.3. Quality assessment

The quality of included articles was independently assessed by 2 authors (KCT and YCW) using the Newcastle-Ottawa scale, with 7 to 9 points indicating high, 4 to 6 indicating moderate, and 0 to 3 indicating low quality (Tables S1 and S2, Supplemental Digital Content, https://links.lww.com/MD/R35). Disagreements were resolved by discussion. For single-arm self-control trials, quality was evaluated using the Murad et al tool for case series,^[18]^ which examines selection, ascertainment, causality, and reporting. Studies satisfying all domains were rated “good” 3 domains “fair” and 2 or fewer domains “poor” (Table S3, Supplemental Digital Content, https://links.lww.com/MD/R35).

### 2.4. Outcomes

In terms of benefits, the main outcomes include the composite outcome of all-cause mortality and HHF, and the separated outcome of HHF and all-cause mortality. The effect of ARNi on LVEF, blood pressure, the level of NT-proBNP, and remodeling of left heart camber (e.g., end diastolic diameters of left ventricular, diameter of left atrium) were also interesting outcomes. For adverse events, the primary outcomes of interest were the occurrence of hyperkalemia, hypotension, and progression to dialysis. The occurrence of outcomes over time was specified according to the available data. Where necessary, the authors of the included articles were contacted by e-mail to obtain additional data.

### 2.5. Statistical analysis

Analyses were performed in RevMan 5.4. Dichotomous outcomes (e.g., mortality, HHF) were expressed as odds ratios (ORs) and continuous outcomes (e.g., LVEF, SBP) as mean differences, both with 95% confidence intervals (CIs). A random-effects model was employed, given the expected heterogeneity, assessed via the *I*^2^ statistic. Statistical significance was set at 5%. Adjusted estimates from each study were pooled with the generic inverse variance method. Publication bias was examined using funnel plots and the Egger regression test. Sensitivity analyses excluded individual studies sequentially.

## 3. Results

### 3.1. Outcomes of literature search and included patients

**Figure 1.**
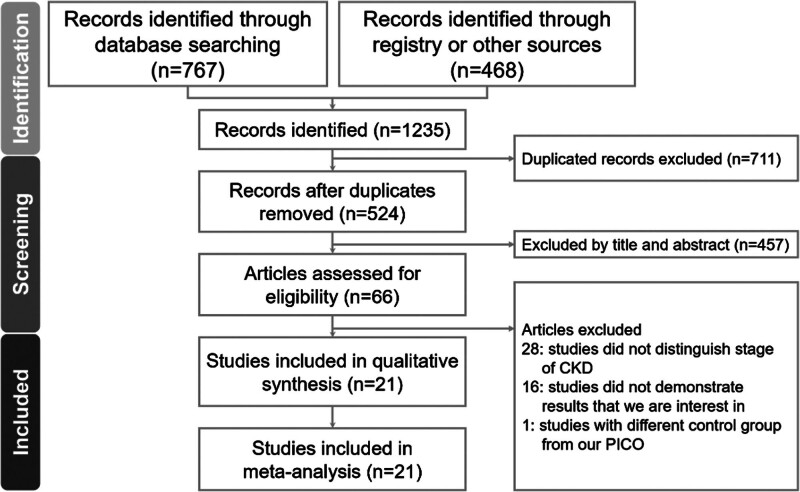
Flow chart for study selection. CKD = chronic kidney disease.

We identified 767 studies from PubMed, Embase, Cochrane, and ClinicalTrials.gov, plus 468 records from other sources. After removing 711 duplicates, 457 were excluded by title/abstract screening. Of the remaining 66 studies, 28 lacked relevant renal subgroup data, 16 did not report our target outcomes, and 1 had an unsuitable control group. Ultimately, 21 articles met the inclusion criteria and are included in this study^[19–39]^ (Fig. 1).

### 3.2. Study characteristics

The enrolled articles include 11 self-control studies and 10 observational studies. The baseline characteristics of included studies are summarized in Table 1. The publication year of the enrolled studies is between 2019 and 2023.

**Table 1:** Characteristics of the enrolled studies.

Author	Publication year	Population		RRT modality	Male	Number (ARNi/ARB)	Quality	Study design	Duration of follow-up
		Renal	Comorbidity						
Lee et al^[20]^	2020	ESRD	HFrEF	HD	87%	23	Fair	Retrospective	132 d
								Self-control	
Fu et al^[21]^	2021	ESRD	HFpEF	PD	66.7%	21	Fair	Retrospective	3–12 mo
								Self-control	
Guo et al^[22]^	2022	ESRD	HFpEF	HD	62.3%	247	Fair	Retrospective	3–12 mo
								Self-control	
Zhang et al^[23]^	2022	ESRD	HFpEF	PD	59.6%	47	Fair	Retrospective	7 d
								Self-control	
Lihua et al^[24]^	2021	ESRD	HFrEF	HD	59%	110	Fair	Retrospective	12 mo
								Self-control	
Iwashima et al^[25]^	2022	ESRD	HTN	HD	69.6%	23	Fair	Retrospective	3 mo
								Self-control	
Wen et al^[26]^	2022	ESRD	HF with LVEF < 55%	HD	53.7%	54	Fair	Retrospective	6 mo
								Self-control	
Pimenta et al^[27]^	2023	ESRD	HFrEF	PD	100%	5	Fair	Retrospective	16 mo
								Self-control	
Cairns et al^[28]^	2023	ESRD	HFrEF	HD/PD	60%	10	Fair	Retrospective	267 d
								Self-control	
Fu et al^[29]^	2023	ESRD	HFpEF	HD	Not mentioned	120	Fair	Prospective	13 mo
								Self-control	
Santhakumari et al^[30]^	2019	ESRD	HFrEF	HD	55%	20	Fair	Prospective	6 mo
								Self-control	
Niu et al^[31]^	2022	ESRD	HFrEF	HD/PD	65.3%	49 (26/23)	8	Prospective	12 mo
								Case control	
								ARNi vs no ARNi	
Hsiao et al^[19]^	2022	Advanced CKD and ESRD	HFrEF	HD	67.5%	1039 (206/833)	9	Retrospective	6.9 ± 4.2 mo
								Case control	
								ARNi vs ACEi/ARB	
Sheng et al^[32]^	2023	ESRD	HFpEF	PD	40%	160 (80/80)	8	Retrospective	6 mo
								Case control	
								ARNi vs no ARNi	
Zhao et al^[33]^	2022	ESRD	PH	HD	61.4%	122 (71/51)	8	Retrospective	3 mo
								Case control	
								ARNi vs ARB	
Ding et al^[34]^	2023	ESRD	–*	HD/PD	68.6%	102 (51/51)	9	Retrospective	349 d
								Case control	
								ARNi vs no ARNi	
Ma et al^[35]^	2023	ESRD	HFpEF	PD	67.7%	99 (61/38)	8	Retrospective	12 mo
								Case control	
								ARNi vs ARB	
Liu et al^[36]^	2023	ESRD	HFrEF and HFpEF	HD/PD	53.4%	116 (67/49)	8	Retrospective	12 mo
								Case control	
								ARNi vs no RAASi	
Chen et al^[37]^‡	2021	Advanced CKD and ESRD	HFrEF	–†	70.6%	1684 (910/774)	9	Retrospective	11.8 ± 12.3 mo
								Cohort	
								ARNi vs ACEi/ARB	
Gula et al^[38]^	2021	ESRD	HFrEF	HD/PD	62%	96 (48/48)	7	Retrospective	3 mo
								Cohort	
								ARNi vs ARB	
Chang et al^[39]^	2023	Advanced CKD and ESRD	HFrEF	Not mentioned	60.9%	510 (232/278)	9	Retrospective	12 mo
								Cohort	
								ARNi vs no ARNi	

ACEi = angiotensin-converting enzyme inhibitor, ARB = angiotensin II receptor blocker, CKD = chronic kidney disease, ESRD = end-stage renal disease, HD = hemodialysis, HFpEF = heart failure with preserved ejection fraction, HFrEF = heart failure with reduced ejection fraction, LVEF = left ventricular ejection fraction, PD = peritoneal dialysis, RAASi = renin–angiotensin–aldosterone system inhibitor, RRT = renal replacement therapy.

* Enroll patients once they have an underlying disease with end-stage disease.

† The study does not mention the RRT modality.

‡ Data extracted from subgroup analysis of renal function.

### 3.3. Self-control studies

Among those self-control studies, all included patients with ESRD, with 10 studies also encompassing patients diagnosed with heart failure. Only 1 study included patients with hypertension. The duration of the follow-up period ranged from 3 to 16 months. The number of patients involved in the studies varied, ranging from 5 to 247 individuals. Regarding KRT modality, 7 of the studies recruited participants exclusively undergoing hemodialysis, while 3 studies enrolled patients receiving peritoneal dialysis. Only 1 study included patients undergoing concomitant hemodialysis and peritoneal dialysis.

### 3.4. Observational studies

Among those observational studies, 7 articles included populations with pure ESRD, while 3 articles included patients with advanced CKD and ESRD. Eight studies encompassed patients with heart failure, with 5 studies investigating patients with reduced ejection fraction and 2 studies discussing those with preserved ejection fraction. There’s 1 study assessing the population of heart failure with a wide range of ejection fractions. One study included patients with comorbid pulmonary hypertension. The duration of the follow-up period ranged from 3 to 12 months, and the number of patients included in the studies varied, ranging from 49 to 1684 individuals.

### 3.5. Quality of enrolled studies and publication bias

The quality of the included studies was deemed high according to the Newcastle-Ottawa scale tool (median score, 8; range from 7–9; Tables S1 and S2, Supplemental Digital Content, https://links.lww.com/MD/R35). There was no significant publication bias in all 4 studies according to Egger weighted regression analyses (*P* = .3244). The quality of the enrolled self-control studies was all classified as fair quality, because questions 6 and 7 were not suitable to evaluate the enrolled studies (Table S4, Supplemental Digital Content, https://links.lww.com/MD/R35).

### 3.6. Primary outcome: composite outcome of hospitalization for heart failure and all-cause mortality, HHF, and all-cause mortality among patients with advanced CKD and ESRD

**Figure 2.**
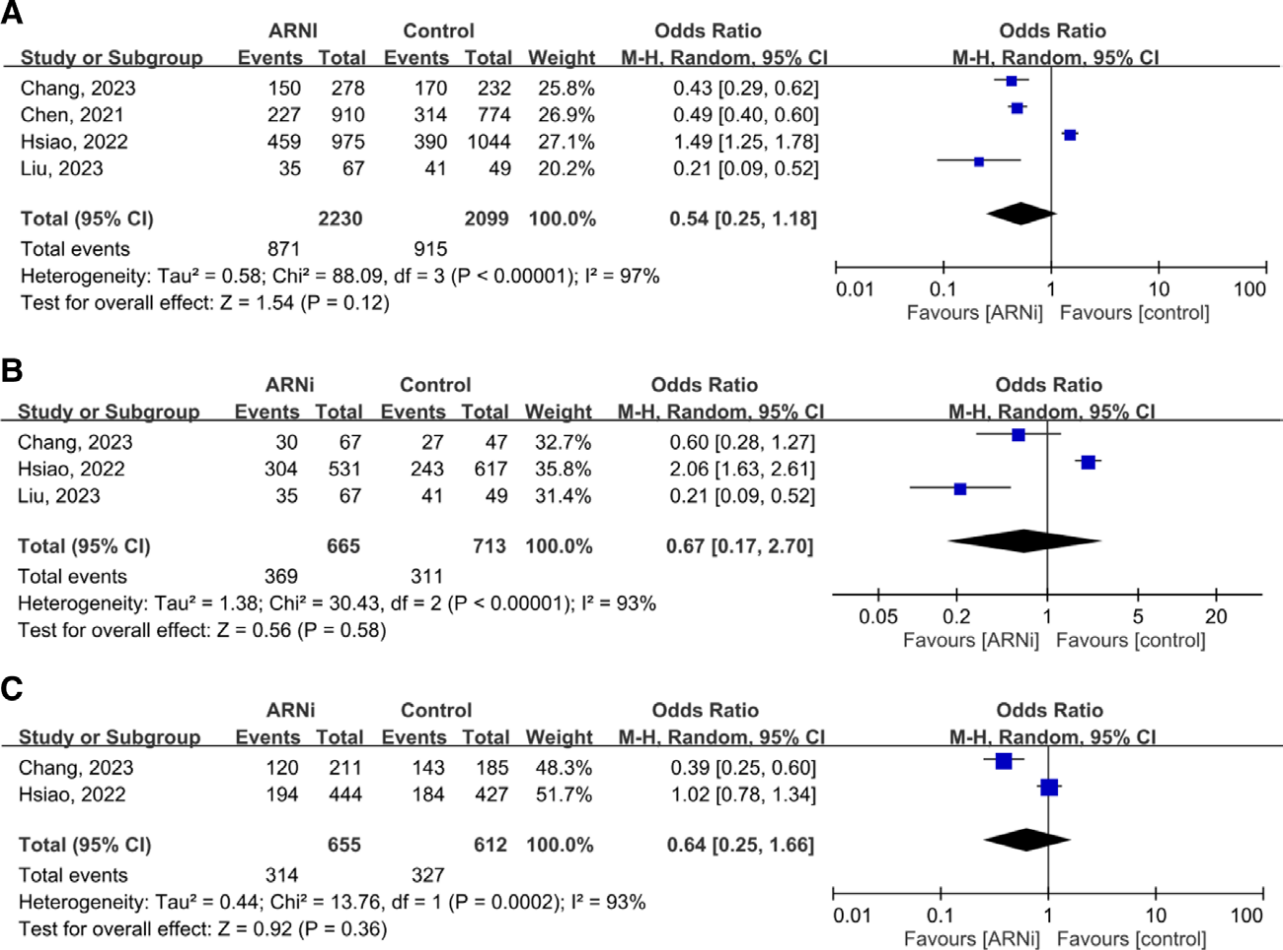
Forest plots comparing the composite outcome in patients with (A) advanced CKD and ESRD, (B) ESRD, and (C) advanced CKD. ARNi = angiotensin receptor neprilysin inhibitor, CI = confidence interval, CKD = chronic kidney disease, ESRD = end-stage renal disease.

**Figure 3.**
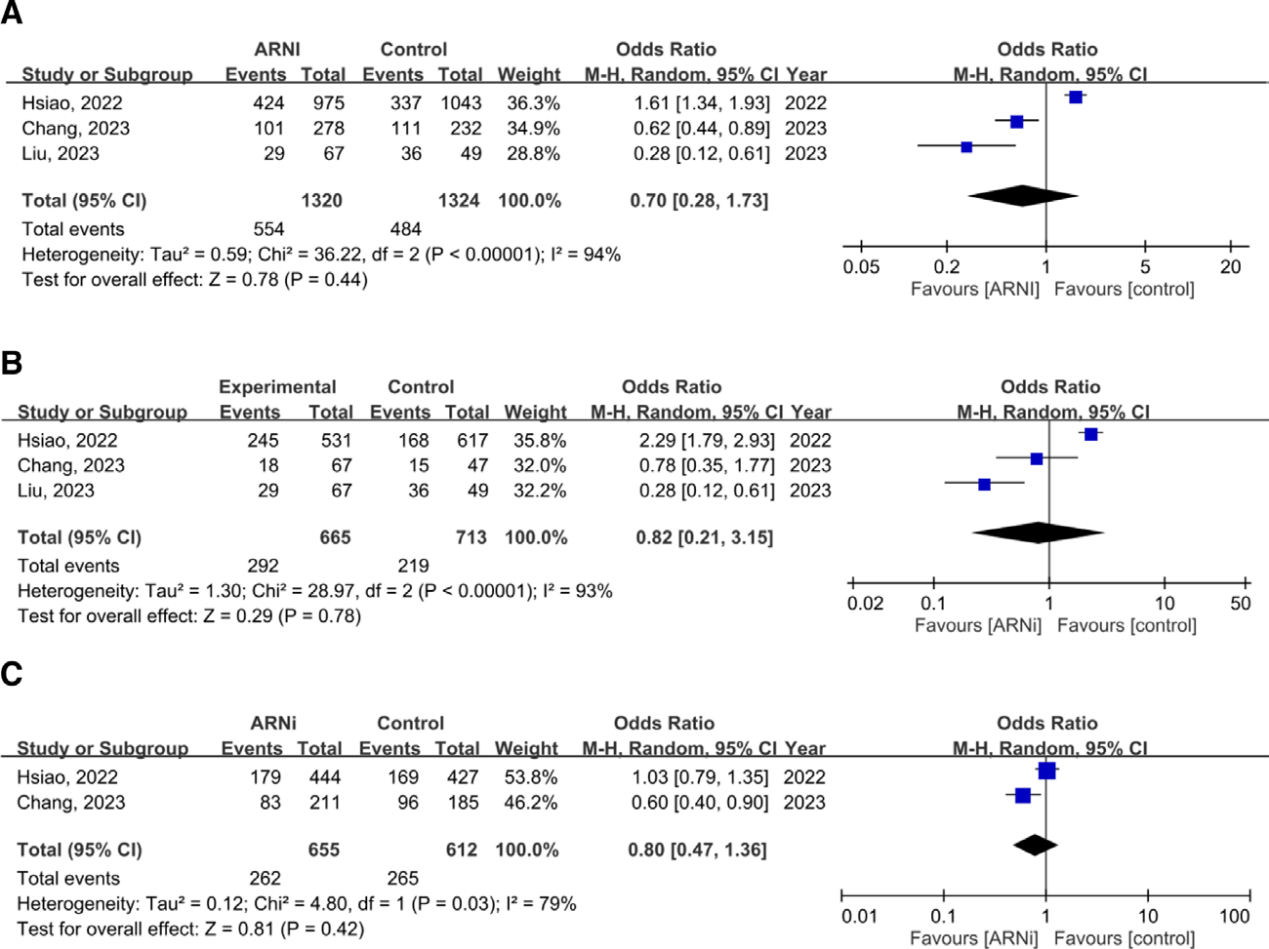
Forest plots comparing the risk of hospitalization for heart failure in patients with (A) advanced CKD and ESRD, (B) ESRD, and (C) advanced CKD. ARNi = angiotensin receptor neprilysin inhibitor, CI = confidence interval, CKD = chronic kidney disease, ESRD = end-stage renal disease.

**Figure 4.**
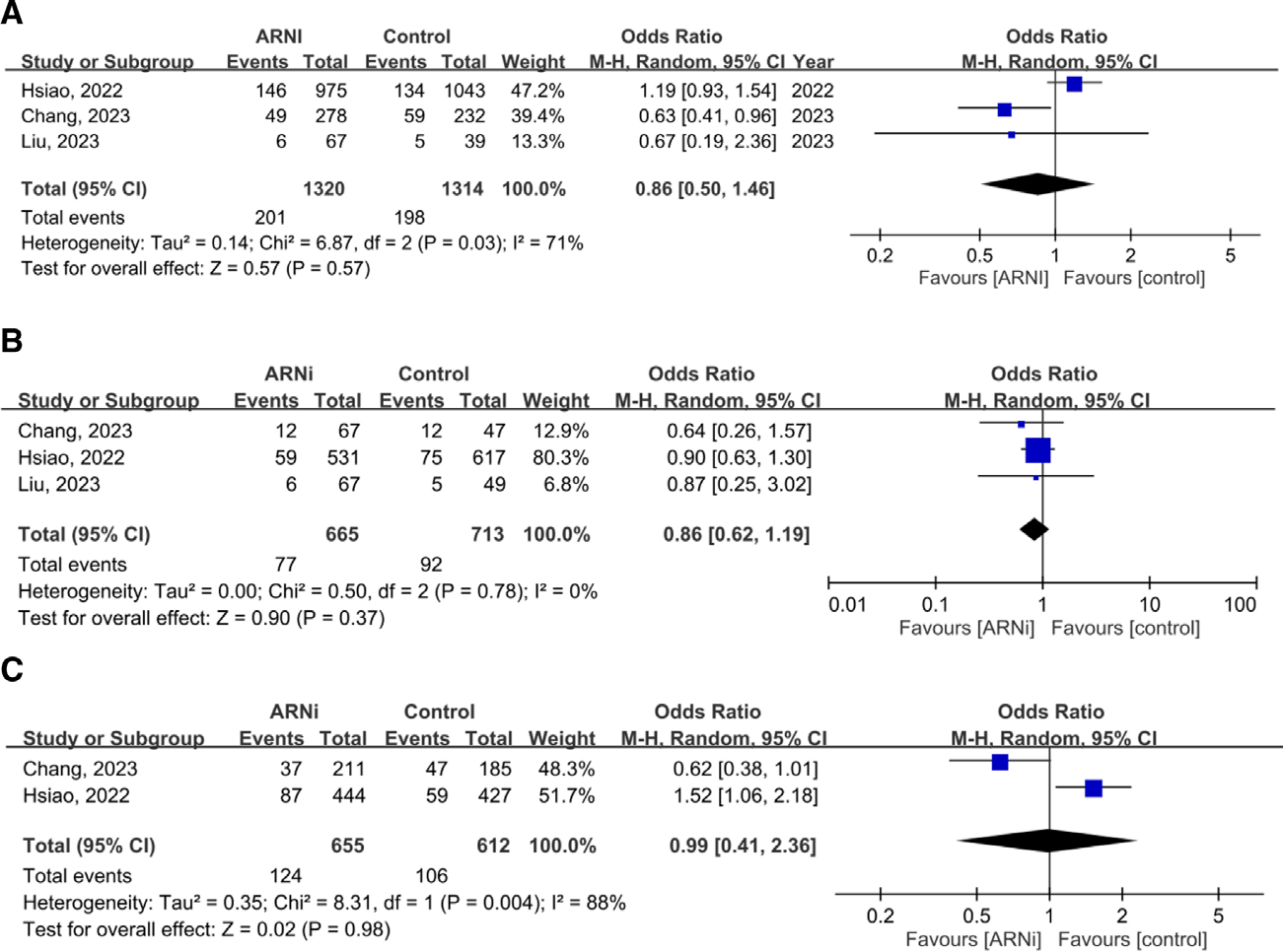
Forest plots comparing all-cause mortality in patients with (A) advanced CKD and ESRD, (B) ESRD, and (C) advanced CKD. ARNi = angiotensin receptor neprilysin inhibitor, CI = confidence interval, CKD = chronic kidney disease, ESRD = end-stage renal disease.

In 4 studies (4329 patients with advanced CKD or ESRD), the pooled OR for the composite outcome (HHF and all-cause mortality) was 0.54 (95% CI: 0.25–1.18, *P* = .12), showing no significant advantage for ARNi. Subgroup analysis for ESRD (OR 0.67, *P* = .58) and CKD (OR 0.64, *P* = .36) yielded similar findings (Fig. 2). Pooled HHF data (three studies, 2644 patients) showed no benefit (OR 0.70, *P* = .44; Fig. 3). For all-cause mortality, the overall pooled OR was 0.86 (*P* = .57), with similarly neutral results in separate CKD and ESRD analyses (Fig. 4). Additionally, the generic inverse variance method was performed to getting the similar result (hazard ratio, 0.91, *P* = .56; Figure S1, Supplemental Digital Content, https://links.lww.com/MD/R35).

### 3.7. Secondary outcome: variation of LVEF, blood pressure, NT-proBNP, LVEDD, and LAD between ARNi and control groups in ESRD patients

**Figure 5.**
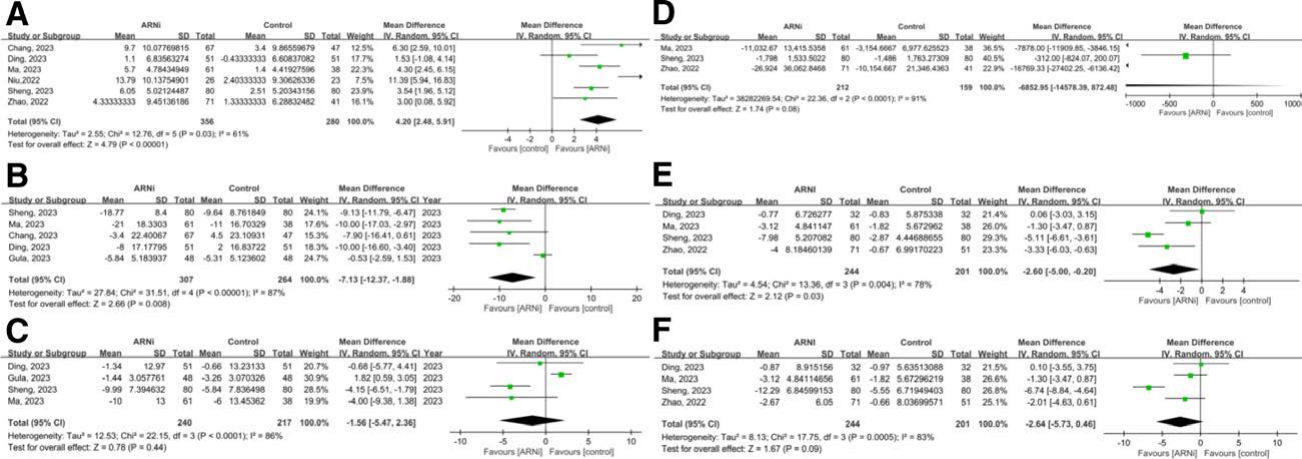
Forest plots comparing pre- and posttreatment metric variations between groups in patients with ESRD: (A) LVEF, (B) SBP, (C) DBP, (D) NT-proBNP, (E) LAD, and (F) LVEDD. ARNi = angiotensin receptor neprilysin inhibitor, CI = confidence interval, DBP = diastolic blood pressure, ESRD = end-stage renal disease, LAD = left atrial diameter, LVEDD = left ventricular end-diastolic dimension, LVEF = left ventricular ejection fraction, NT-proBNP = N-terminal pro–B-type natriuretic peptide, SBP = systolic blood pressure.

In 6 studies (636 patients), ARNi improved LVEF by 4.20% (95% CI: 2.48–5.91, *P* < .001). Five studies showed a mean SBP reduction of 7.13 mm Hg (*P* = .008), but DBP remained unchanged. ARNi did not significantly lower NT-proBNP (−6853, *P* = .08). Regarding remodeling, LVEDD decreased by −2.64 mm (*P* = .09), while LAD showed a modest reduction of −2.60 mm (*P* = .03; Fig. 5).

### 3.8. Safety outcome: the risk of hyperkalemia and progression to ESRD in CKD patients

**Figure 6.**
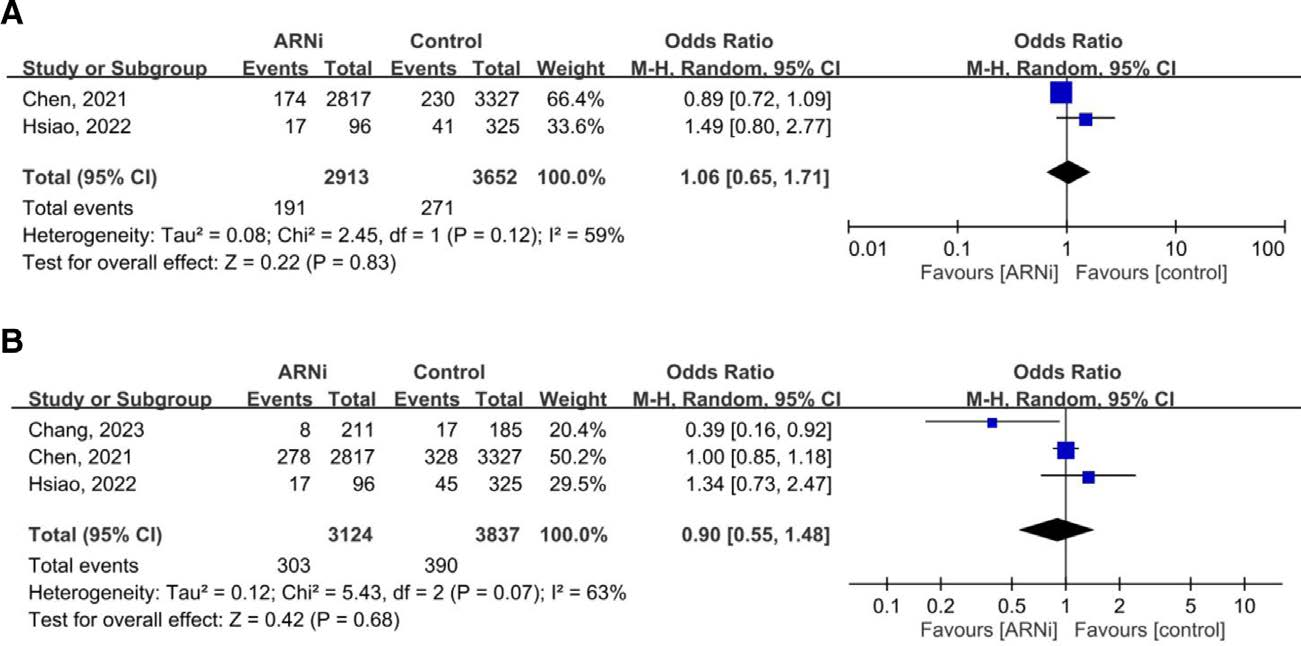
Forest plots comparing the risk of hyperkalemia (A) and progression to ESRD (B) in CKD patients. ARNi = angiotensin receptor neprilysin inhibitor, CI = confidence interval, CKD = chronic kidney disease, ESRD = end-stage renal disease.

ARNi does not appear to be associated with an increased risk of hyperkalemia in advanced CKD patients when compared to control, as indicated by the pooled OR from 2 studies, which is 1.06 (95% CI: 0.65–1.71, *P* = .83). Furthermore, after pooling data from 3 articles, ARNi was also not associated with an elevated risk of progression to ESRD in advanced CKD patients when compared to control groups, with a pooled OR of 0.90 (95% CI: 0.55–1.48; Fig. 6).

### 3.9. Safety outcome: the risk of hyperkalemia and hypotension in ESRD patients

**Figure 7.**
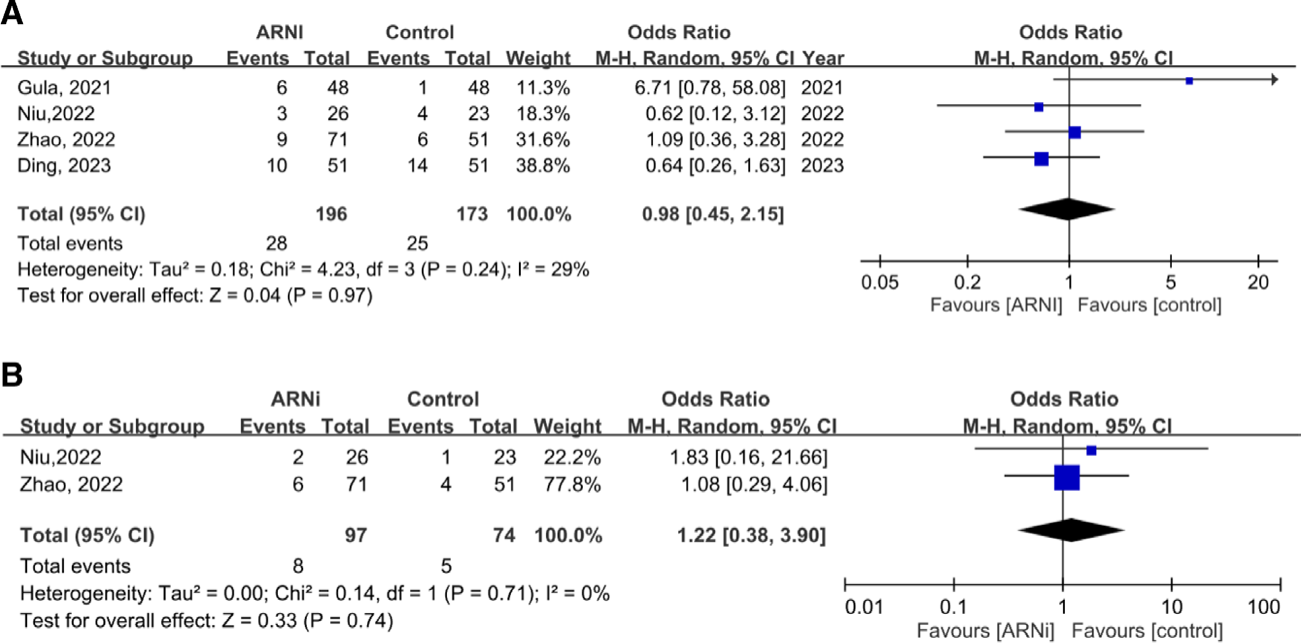
Forest plots comparing the risk of hyperkalemia (A) and hypotension (B) in ESRD patients. ARNi = angiotensin receptor neprilysin inhibitor, CI = confidence interval, ESRD = end-stage renal disease.

The pooled OR regarding the risk of hyperkalemia in ESRD patients, based on data from 4 studies involving 369 patients, is 0.98 (95% CI: 0.45–2.15, *P* = .97), indicating that ARNi did not increase the risk of hyperkalemia. On the other hand, concerning hypotension, the pooled OR from 2 studies is 1.22, but it is not statistically significant (95% CI: 0.38–3.90, *P* = .74; Fig. 7).

### 3.10. Secondary outcome: variation of LVEF, blood pressure, NT-proBNP, LVEDD, and LAD after using ARNi in ESRD patients from self-control studies

**Figure 8.**
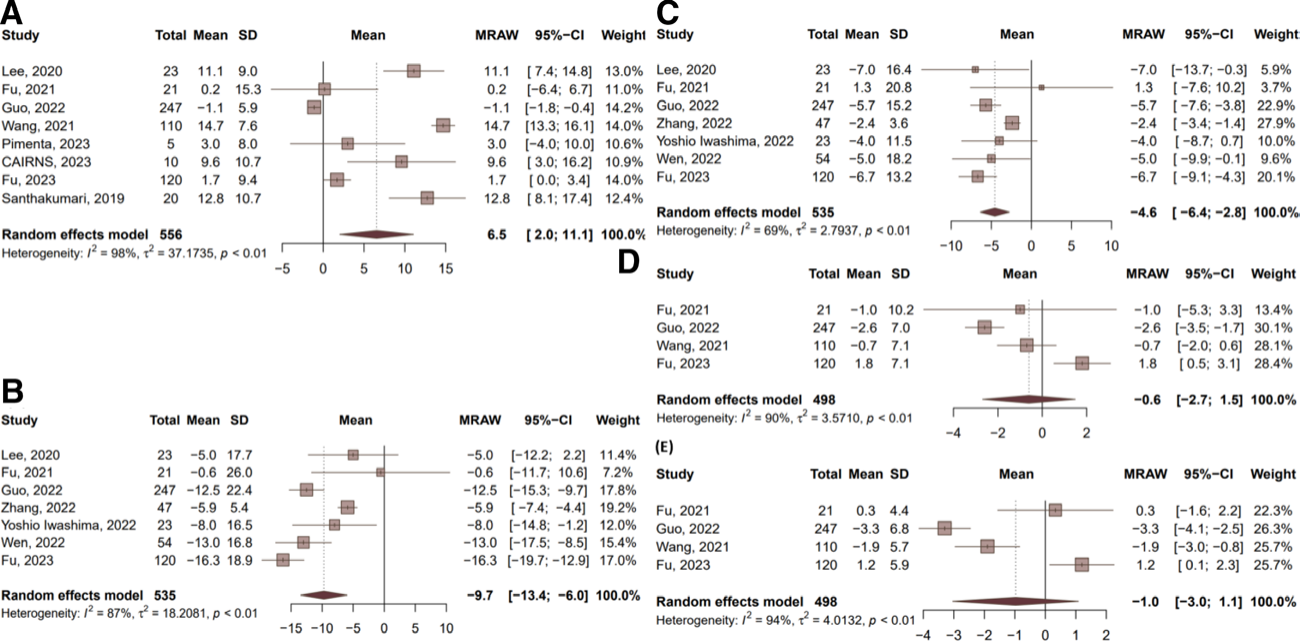
Pre- and posttreatment metrics in patients with ESRD from self-control studies. (A) LVEF, (B) SBP, (C) DBP, (D) LVEDD, (E) LAD. CI = confidence interval, DBP = diastolic blood pressure, ESRD = end-stage renal disease, LAD = left atrial diameter, LVEDD = left ventricular end-diastolic dimension, LVEF = left ventricular ejection fraction, SBP = systolic blood pressure.

In 8 self-control ESRD studies, ARNi improved LVEF by 6.5% (95% CI: 2.0–11.1). Seven studies (535 patients) showed a mean SBP reduction of 9.7 mm Hg and a DBP reduction of 4.6 mm Hg. From 5 studies, NT-proBNP decreased by 10,250.7 (95% CI: −16,908 to −3593). LVEDD and LAD showed minimal changes. All results exhibited high heterogeneity (Fig. 8).

### 3.11. Sensitivity analysis

**Figure 9.**
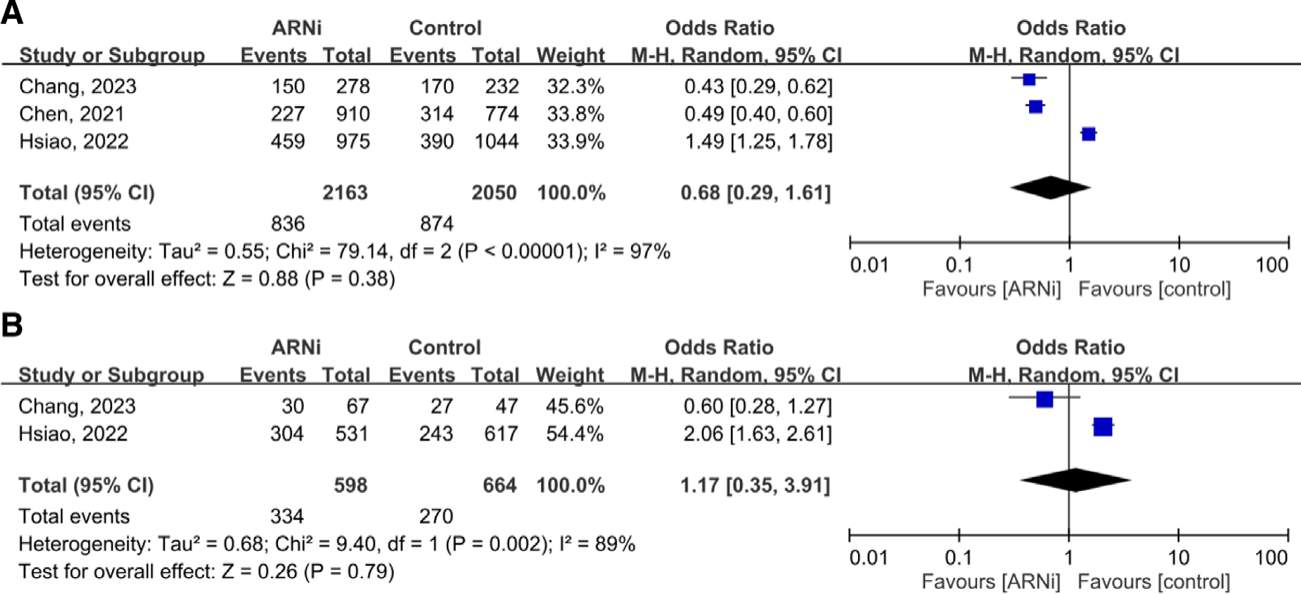
Forest plots of sensitive analysis for comparing the composite outcome in patients with (A) advanced CKD and ESRD, (B) ESRD. ARNi = angiotensin receptor neprilysin inhibitor, CI = confidence interval, CKD = chronic kidney disease, ESRD = end-stage renal disease.

We excluded Liu et al^[36]^ (due to unmatched groups) and obtained similar findings. The pooled OR for advanced CKD and ESRD was 0.68 (95% CI: 0.29–1.61), and for ESRD alone, 1.17 (95% CI: 0.35–3.91; Fig. 9).

## 4. Discussion

The primary outcome of the current meta-analysis, which included 4 observational studies, indicated that ARNi did not exhibit a statistically significant reduction in the OR for HHF and all-cause mortality in the advanced CKD and ESRD group. Within the CKD group, ARNi had a neutral effect on HHF and all-cause mortality when compared to the control group. Similarly, within the ESRD group, the advantageous effects of ARNi on HHF and all-cause mortality were also not observed, and the pooled data only showed a non-statistically significant lower OR concerning HHF and all-cause mortality when compared with the control groups. On the other hand, data pooled from 11 self-control studies revealed that ESRD patients using ARNi may experience several benefits, including improvements in LVEF, reductions in blood pressure, and decreases in the level of N-terminal pro NT-proBNP. Furthermore, these benefits attributed to ARNi were also observed in observational cohort studies, and notably, the advantageous effect appeared to be even more pronounced when compared to control groups.

Natriuretic peptide, which undergoes degradation by neutral endopeptidase-neprilysin, possesses several beneficial effects, including increasing urine and salt excretion, vasodilation, inhibition of the RAAS, and amelioration of cardiac remodeling. The excretion of valsartan is mainly from the biliary route, and the renal function did not affect it much. Sacubitrilat, the active form for inhibition of neprilysin converted from sacubitril, is eliminated mainly from the kidney. This implies that its exposure in the body may increase as renal function declines, as the impaired kidneys may have a reduced capacity to clear sacubitrilat from the system.^[17]^ Theoretically, in patients with advanced CKD, the effect of ARNi could potentially offer cardiovascular benefits as the blood concentration of the active form of the drug (sacubitrilat) is increased due to reduced renal clearance. This increased exposure to sacubitrilat might enhance its ability to inhibit neprilysin, leading to greater natriuretic peptide levels and the associated cardiovascular benefits, such as improved fluid balance, vasodilation, and cardiac remodeling.^[40]^ Although some studies have investigated the effects of ARNi in patients with CKD regarding cardiovascular protection and renal function,^[41,42]^ this discussion primarily focuses on patients with an eGFR >30 mL/min/1.73 m^2^. Most large-scale RCTs have predominantly excluded patients with an eGFR <30 mL/min/1.73 m^2^. Only the HARP III trial included patients with CKD stage 4, revealing that ARNi has a similar effect on renal protection as angiotensin II receptor blocker (ARB) and can further improve blood pressure and NT-proBNP levels when compared with ARB.^[43]^ A real-world study in Taiwan reported that advanced CKD (CKD 4 and 5) patients treated with ARNi had a 28% lower risk of cardiovascular death and HHF when compared to those receiving standard heart failure care, and this effect is more pronounced than in patients with eGFR > 30 (mL/min/1.73 m^2^).^[15]^ However, in Hsiao et al’s study, the impact of ARNi on patients with advanced CKD is not so significant when it comes to HHF and all-cause mortality.^[19]^ The current meta-analysis indicates that, in comparison to the control group, the effect of ARNi on the end point of all-cause mortality and HHF is neutral among patients with concomitant HFrEF and CKD stage 4 and 5.

The characteristics of patients undergoing maintenance dialysis for ESRD are fundamentally different from those with CKD stage 4 and 5 in various aspects, including fluid status, hemodynamic status, hormone homeostasis, uremic symptoms, and even the risk of MACE rate.^[44]^ Due to the absence of robust RCT evidence to support the effects of ARNi on ESRD patients, there remains controversy regarding whether the effects of ARNi on ESRD patients align with those observed in patients with CKD. Pharmacokinetic studies have shown that patients with ESRD undergoing maintenance dialysis do not efficiently remove valsartan and the active metabolite of sacubitril and sacubitrilat.^[45,46]^ This results in sustained therapeutic plasma concentrations, suggesting that ARNi may remain pharmacodynamically active in the ESRD population. These pharmacokinetic properties may help explain the observed improvements in surrogate endpoints in this meta-analysis. The current study, which aggregated data from 11 self-control studies, supports this hypothesis. It showed consistent improvements in left ventricular systolic function, reductions in NT-proBNP concentrations, and better blood pressure control following ARNi treatment in patients on maintenance dialysis. These findings were further corroborated by observational cohort studies. Importantly, ARNi use in this population was not associated with a significant increase in adverse events, indicating a favorable safety profile. While improvements in surrogate markers are encouraging, the neutral findings in terms of HHF and all-cause mortality underscore the need for prospective, randomized trials to clarify the long-term clinical benefits of ARNi in the ESRD population.

In Hsiao et al’s study, an increasing risk between ARNi usage and composite outcome of HHF and all-cause mortality is observed in patients with advanced CKD and ESRD, and this trend is mainly influenced by those with ESRD and the result of HHF.^[19]^ The reason for this finding, they explained, is that ARNi could lower the blood pressure more aggressively than angiotensin-converting enzyme inhibitor/ARB, and this would cause more hypotension and less ultrafiltration during dialysis, which leads to an increased risk of HHF. However, in the current meta-analysis, including 2 studies, we found that there’s no increased risk of hypotension in patients with ESRD who undergo ARNi administration when compared to the control group. In contrast, in Chang et al’s^[15]^ study, analysis of 2 registries from Taiwan, including 114 patients with ESRD, revealed that ARNi is not associated with an increased risk of HHF in ESRD patients. Furthermore, in Liu et al’s^[36]^ article, ARNi is associated with less HHF and all-cause mortality in patients with ESRD. But unlike to the former 2 studies, including patients with HFrEF, the latter study enrolled populations with a wide range of ejection fractions. Besides, the small case number and lack of a group-matching process and RAAS inhibitor control group make it inherently different from the former 2 studies. Without the inclusion of other RAAS blockers as comparators, the interpretation of the effect of ARNi on hard clinical endpoints across different stages of renal disease should be approached with caution. To address this limitation, a sensitivity analysis excluding the study in question was performed, which yielded consistent results regarding the risks of HHF and all-cause mortality in patients with ESRD.

In addition to pharmacologic therapy for heart failure in patients with ESRD, device-based interventions such as implantable cardioverter-defibrillators (ICDs) and cardiac resynchronization therapy play a critical role in comprehensive heart failure management.^[47]^ Given the substantially increased risk of sudden cardiac death and arrhythmias among dialysis patients, timely ICD implantation may confer a survival benefit. However, the risk–benefit profile in ESRD is complex due to competing causes of mortality and a higher incidence of device-related complications. Hayiroğlu et al demonstrated that the Multicenter Automatic Defibrillator Implantation Trial II score effectively predicts 1-year and long-term mortality in elderly HFrEF patients with ICDs, highlighting the need for individualized risk stratification.^[48]^ Çinier et al showed that a lower prognostic nutritional index is strongly associated with increased long-term mortality, emphasizing the importance of assessing nutritional and inflammatory status in guiding device therapy.^[49]^ Together, these findings emphasize the value of integrating pharmacologic therapies such as ARNi with personalized device-based strategies in ESRD patients with HFrEF. Considering the altered pharmacokinetics of ARNi in ESRD and the complex risk profiles for ICD and cardiac resynchronization therapy, a comprehensive approach incorporating individualized risk scores and biomarkers like prognostic nutritional index may improve clinical outcomes in this high-risk and underrepresented population.

There are some limitations in the current systematic review and meta-analysis. Firstly, all the included articles are observational studies, and there is evident heterogeneity among the enrolled studies concerning study design, the population under study, and the strategy for KRT. We conducted a sensitivity analysis and adjusted point estimates and standard errors for each study using the generic inverse variance method to eliminate the heterogeneity between studies. Despite these efforts, the pooled results remained consistent. Furthermore, we were unable to perform a subgroup analysis on the effects of ARNi across various KRT modalities due to the lack of detailed data in the original articles. Secondly, some studies only presented diagrams without exact data. To address this, we utilized WebPlotDigitizer to extract the necessary data. Thirdly, although the funnel plot and Egger test indicated low risk of publication bias in the enrolled studies, the limited number of studies (<10) might not have enough statistical power to meet the assumption. Fourthly, the assessment of self-control studies was conducted using the method proposed by Murad et al.^[18]^ However, the evaluation questions in the causality domain, including 2 questions focusing on drug adverse effects, may not be suitable for adequately evaluating the enrolled studies.

## 5. Conclusion

The study did not demonstrate a significant association between ARNi use and reductions in HHF or all-cause mortality. However, we observed a potential improvement in LVEF among HFrEF patients with ESRD, suggesting some benefits of ARNi. Notably, using ARNi in the ESRD group does not appear to increase the risks of hyperkalemia or hypotension. Given the inherent limitations in establishing causality in observational studies, there is a pressing need for RCTs to conclusively evaluate the effectiveness and safety of ARNi in this patient population.

## Author contributions

**Conceptualization:** Kuan-Chieh Tu, Yu-Chieh Weng, Jheng-Yan Wu, Chia-Te Liao.

**Methodology:** Jheng-Yan Wu.

**Data curation:** Yu-Chieh Weng.

**Formal analysis:** Yu-Chieh Weng.

**Software:** Jheng-Yan Wu.

**Writing – original draft:** Kuan-Chieh Tu, Yu-Chieh Weng, Jheng-Yan Wu, Chia-Te Liao.

**Writing – review & editing:** Jheng-Yan Wu, Chia-Te Liao.

## Supplementary Material


