# Supplementary material for: The effectiveness and safety of sacubitril/valsartan in real-world dialysis patients with heart failure reduced ejection fraction
Source: Medicine (Baltimore). 2026 Jan 30;105(5):e46866. doi: 10.1097/MD.0000000000046866 (PMC12863774; doi:10.1097/MD.0000000000046866)
Supplement: Supplementary file 1 [file medi-105-e46866-s001.docx]

|  | **Selection** | | | | **Comparability** |  | **Exposure** | |  |
| --- | --- | --- | --- | --- | --- | --- | --- | --- | --- |
| First author / Year | **Is the Case Definition Adequate** | **Representativeness of the Cases** | **Selection of Controls** | **Definition of Controls** | **Comparability of Cases and Controls on the Basis of the Design or Analysis** | **Ascertainment of Exposure** | **Same method of**  **ascertainment for**  **cases and controls** | **Non-Response Rate** | **Total** |
| Niu/2022 | * | * | * | * | * | * | * | * | 8 |
| Hsiao/2022 | * | * | * | * | ** | * | * | * | 9 |
| Ding/2023 | * | * | * | * | ** | * | * | * | 9 |
| Zhao/2022 | * | * | * | * | * | * | * | * | 8 |
| Ma/2023 | * | * | * | * | * | * | * | * | 8 |
| Liu/2023 | * | * | * | * | * | * | * | * | 8 |
| Sheng/2023 | * | * | * | * | * | * | * | * | 8 |

**S1 Table. Newcastle-Ottawa Scale Quality Assessment of included studies (case-control study)**

|  | **Selection** | | | | **Comparability** |  | **Exposure** | |  |
| --- | --- | --- | --- | --- | --- | --- | --- | --- | --- |
| First author / Year | **Representativeness of the Exposed Cohort** | **Selection of the Non-Exposed Cohort** | **Ascertainment of Exposure** | **Demonstration That Outcome of Interest Was Not Present at Start of Study** | **Comparability of Cohorts on the Basis of the Design or Analysis** | **Assessment of Outcome** | **Was Follow-Up Long Enough for Outcomes to Occur** | **Adequacy of Follow Up of Cohorts** | **Total** |
| Chang/2023 | * | * | * | * | ** | * | * | * | 9 |
| Chen/2021 | * | * | * | * | ** | * | * | * | 9 |
| Gula/2021 | * | * | * | * | * | * | * | 0 | 7 |

**S2 Table. Newcastle-Ottawa Scale Quality Assessment of included studies (cohort study)**

| **Domains** | **Leading explanatory questions** |
| --- | --- |
| Selection | 1. Does the patient represent the whole experience of the investigator (center) or is the selection method unclear to the extent that other patients with similar presentation may not have been reported? |
| Ascertainment | 2. Was the exposure adequately ascertained? |
|  | 3. Was the outcome adequately ascertained? |
| Causality | 4. Were other alternative causes that may explain the observation ruled out? |
|  | 5. Was there a challenge and/or rechallenge phenomenon? |
|  | 6. Was there a dose-response effect? |
|  | 7. Was follow-up long enough for outcomes to occur? |
| Reporting | 8. Is the case described with sufficient details to allow other investigators to replicate the research or to allow practitioners to make inferences related to their own practice? |

**S3 Table: Tool used for the evaluation of the methodological quality of case reports and case series**

**
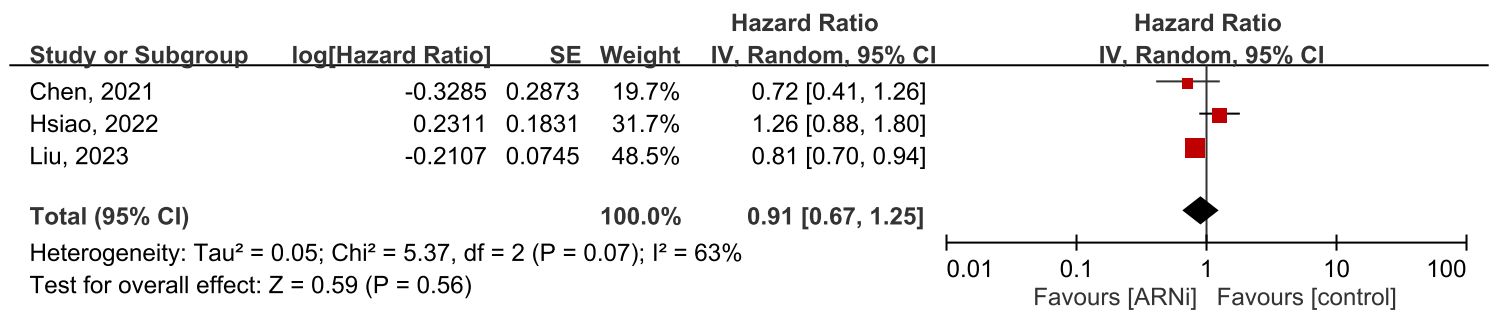
**

**Figure S1. Forest plot of** **generic inverse variance method for comparing the composite outcome in patients with advanced CKD and ESRD**
